# Cross-cultural adaptation and psychometric validation of the breast cancer stigma assessment scale among Chinese patients

**DOI:** 10.3389/fpsyg.2025.1641611

**Published:** 2025-11-19

**Authors:** Youbei Lin, Chuang Li, Hongyu Li, Xiuli Wang

**Affiliations:** 1 Department of Cancer Clinical Research Ward, The First Affiliated Hospital of Jinzhou Medical University, Jinzhou, China; 2 School of Nursing, Jinzhou Medical University, Jinzhou, China

**Keywords:** breast cancer, stigma, cross-cultural adaptation, psychometric validation, confirmatory factor analysis

## Abstract

**Background:**

Breast cancer stigma significantly impacts patients’ psychological wellbeing, yet culturally validated assessment tools remain limited in Chinese contexts. This study aimed to translate and culturally adapt the Breast Cancer Stigma Assessment Scale (BCSAS) and evaluate its psychometric properties among Chinese breast cancer patients.

**Methods:**

Following Brislin’s translation model, the BCSAS was rigorously adapted through forward-backward translation and cultural adaptation. Six multidisciplinary experts (nursing psychology, *n =* 4; breast surgery, *n =* 2) evaluated content, semantic, and conceptual equivalence. A total of 550 questionnaires were distributed to women with breast cancer from three tertiary hospitals in western Liaoning, China, yielding 500 valid responses (response rate = 90.91%). Psychometric evaluation included content validity assessment, confirmatory factor analysis (CFA), reliability testing, and convergent validity assessment. Exploratory network analysis complemented CFA findings.

**Results:**

The Chinese version (C-BCSAS) demonstrated excellent content validity (S-CVI = 0.98), strong internal consistency (Cronbach’s *α* = 0.890; dimension-specific *α* = 0.712–0.876), and good test–retest reliability (ICC = 0.825, 95% CI = 0.691–0.903). CFA revealed fit indices of *χ*^2^/df = 4.446, CFI = 0.829, TLI = 0.800, RMSEA = 0.083. While slightly below commonly cited thresholds, all factor loadings substantially exceeded 0.50 (range: 0.540–0.846, *p <* 0.001), supporting item-level validity. The original seven-factor, 28-item structure was retained to preserve theoretical integrity and enable cross-cultural comparisons.

**Conclusion:**

The C-BCSAS is a reliable and culturally valid instrument for assessing breast cancer stigma in Chinese contexts, suitable for both clinical assessment and international comparative research.

## 1 Introduction

Breast cancer is a malignant tumor originating from breast tissue and is one of the most common cancers among women worldwide, accounting for approximately 25% of all female cancers (Lei et al., 2021). According to the latest report from the National Cancer Center of China, breast cancer ranks second among malignancies in Chinese women, with 357,200 newly diagnosed cases in 2022 (Sun et al., 2024). The incidence continues to rise annually, and the age of onset is trending younger. With the widespread implementation of early screening and advances in comprehensive treatment strategies, survival rates of breast cancer patients have improved substantially (Tang et al., 2025); however, the psychosocial challenges associated with the disease and its treatment have become increasingly prominent (Yang et al., 2025). Among these psychosocial challenges, stigma has emerged as a particularly salient issue, as patients must not only cope with physical sequelae of treatment but also navigate negative societal perceptions attached to the illness (Zamanian et al., 2022).

Stigma, first conceptualized by Goffman (1963), refers to the social process whereby individuals are negatively evaluated and marginalized due to certain attributes (Goffman, 1963). Weiss et al. (2006) extended this conceptualization to health contexts, proposing a comprehensive framework that identifies six key dimensions of health-related stigma: enacted stigma (experienced discrimination), felt/internalized stigma (shame and self-devaluation), disclosure concerns, negative self-image, and concerns about public attitudes (Weiss et al., 2006). Health-related stigma encompasses feelings of shame, guilt, or embarrassment associated with illness, often shaped by societal negative perceptions (Stangl et al., 2019). Originally applied to mental health and infectious diseases, stigma theory has since been extended to chronic conditions and cancer (Wu et al., 2023), where it operates through similar mechanisms of social devaluation, concealment, and identity threat. Health-related stigma can lead to delayed medical consultation, symptom concealment, poor treatment adherence, and ultimately unfavorable health outcomes (Stangl et al., 2019).

Stigma among breast cancer patients is distinctive. Treatment-related physical changes (mastectomy, alopecia, lymphedema) can heighten patients’ sense of difference from healthy individuals, disrupting body image and affecting self-identity and social participation (An et al., 2022; Bu et al., 2022; Wu et al., 2021). Stigma contributes to lower self-esteem, psychological distress, social avoidance, and compromised quality of life (Pham et al., 2021; Stangl et al., 2019), underscoring the clinical importance of accurate assessment and intervention.

Several instruments have been developed to assess cancer-related stigma, including generic tools such as the Social Impact Scale (SIS)(Fife and Wright, 2000) and the Cataldo Cancer Stigma Scale (CCSS)(Cataldo et al., 2011), as well as disease-specific measures like the Breast Cancer Stigma Scale (BCSS) (Bu et al., 2022). However, systematic reviews conducted under COSMIN guidelines have shown that most existing instruments were developed within Western cultural contexts and display limited cross-cultural adaptability (Xie et al., 2023). The U. S. National Cancer Institute has also emphasized that stigma varies by culture, contributing to global inequities in cancer care, while research on cancer stigma remains culturally underrepresented (Heley et al., 2024).

In response to these limitations, the Breast Cancer Stigma Assessment Scale (BCSAS) was developed by Cenit-García et al. (2024) using rigorous psychometric methodology to provide a comprehensive measure of breast cancer-related stigma (Cenit-García et al., 2024). The BCSAS is grounded in Fujisawa and Hagiwara’s established cancer stigma framework (Fujisawa and Hagiwara, 2015)and incorporates dimensions that capture both self-perception and social interaction aspects of stigma.

Qualitative research with Chinese breast cancer patients has identified recurring themes of family-centered concerns, including intense worry about burdening family members, disrupting family harmony, and bringing shame to family reputation (Cui et al., 2021; Jin et al., 2021; Li et al., 2023; Xu et al., 2021; Yeung et al., 2019). Importantly, comparative welfare research has documented substantial similarities in family-centered values between Southern European and East Asian “familialistic” cultures, both emphasizing family responsibility and intergenerational obligations over individual autonomy (Saraceno, 2016). The BCSAS includes a Family Disruption dimension that addresses such concerns; however, whether this Western-developed dimension adequately captures the culturally specific manifestations of family-related stigma in Chinese contexts remained an empirical question requiring systematic validation.

Given the cultural sensitivity of stigma assessment and the need for cross-cultural measurement invariance, it is essential to establish a culturally adapted and psychometrically robust tool tailored for Chinese breast cancer patients. Such an instrument would not only provide a reliable measure for clinical screening and intervention evaluation but also deepen understanding of stigma mechanisms within Chinese cultural settings, thereby contributing valuable methodological evidence to the global literature on cancer stigma. The present study aimed to translate and culturally adapt the BCSAS (Cenit-García et al., 2024) into Chinese and to systematically evaluate its psychometric properties, including reliability, content validity, and structural validity, among Chinese women with breast cancer and survivors. This culturally adapted instrument is expected to enable accurate assessment of stigma experiences in Chinese contexts, thereby informing targeted psychological interventions and facilitating cross-cultural stigma research.

## 2 Methods

### 2.1 Study design and ethical approval

This cross-sectional study aimed to translate and psychometrically validate the Breast Cancer Stigma Assessment Scale (BCSAS) (Cenit-García et al., 2024) for Chinese contexts. The research was grounded in Goffman’s stigma theory and Fujisawa and Hagiwara’s cancer stigma framework (Fujisawa and Hagiwara, 2015). Ethical approval was obtained from the Ethics Committee of Jinzhou Medical University (Approval No. JZMULL20240704). All participants provided written informed consent, and the study adhered to the Declaration of Helsinki.

### 2.2 Instrument translation and cultural adaptation

#### 2.2.1 Original scale

The Breast Cancer Stigma Assessment Scale (BCSAS) was developed by Cenit-García et al. (2024) in Spain, based on the theoretical framework of Fujisawa and Hagiwara (Fujisawa and Hagiwara, 2015). The instrument consists of 28 items across seven dimensions: concealability, discrimination, altered self-image/self-concept, family disruption, social attribution, prejudice, and origin. Items are rated on a five-point Likert scale (1 = strongly disagree to 5 = strongly agree), with total scores ranging from 28 to 140. Higher scores indicate greater levels of stigma. The scale was developed using rigorous psychometric methodology. Content validity was evaluated through a two-round Delphi study involving 15 multidisciplinary experts from anthropology, sociology, psychology, oncology, and nursing (mean work experience = 19.8 years), achieving 93.3% consensus (Cenit-García et al., 2024). The original scale demonstrated excellent internal consistency (Cronbach’s *α* = 0.897) and strong test–retest reliability (r = 0.830, *p <* 0.001) (Cenit-García et al., 2024).

#### 2.2.2 Translation and cultural adaptation

Permission for translation was obtained from the original author. The translation process followed Brislin’s model (Hu et al., 2025). Two nursing master’s students independently translated the scale into Chinese (versions T1 and T2), which were synthesized into an initial Chinese version (T3). The research team reviewed and finalized this as the forward-translated version (T-12) (Li et al., 2025a). Two English graduate students, blinded to the original scale, then back-translated T-12 into English (versions B1 and B2) (Li et al., 2025b). Semantic equivalence was compared with the original version, achieving over 90% consistency, and the back-translation was confirmed by the original author (Chang et al., 2024). During cultural adaptation, six experts (four nursing psychology specialists and two breast surgery specialists) evaluated the draft for content, semantic, and conceptual equivalence. Expert inclusion criteria were: (1) mastery of relevant professional knowledge; (2) more than 10 years of work experience (or more than 5 years for those with doctoral degrees); and (3) intermediate or higher professional titles. Items were revised according to their suggestions to ensure cultural relevance and linguistic clarity. Items were revised according to their suggestions to ensure cultural relevance and linguistic clarity. Specific modifications are detailed in Section 3.2.

#### 2.2.3 Pilot testing

A pilot study was conducted in March 2024 at the First Affiliated Hospital of Jinzhou Medical University with 30 breast cancer patients. Pilot testing was conducted with 20–40 participants in line with best-practice guidelines for cognitive interviewing studies, which recommend such sample sizes to reach saturation in identifying comprehension issues (Blair and Conrad, 2011; Guest et al., 2020). Cognitive interviews were used to assess participants’ comprehension of items and instructions (Edelen et al., 2014; Effa et al., 2022). During the interviews, participants were asked to: (1) complete the questionnaire while thinking in a quiet hospital room environment; (2) explain the items in their own words; (3) describe their response choices; and (4) identify any confusing or culturally inappropriate content.

### 2.3 Formal survey

#### 2.3.1 Participants

Participants were recruited between March and September 2024 from the breast surgery and oncology departments of two tertiary hospitals in Jinzhou, China, using convenience sampling. The inclusion criteria were: (1) female patients with pathologically confirmed breast cancer; (2) aged ≥18 years; (3) disease duration ≥3 months; (4) clear consciousness and intact communication skills; (5) ability to understand questionnaire items; and (6) willingness to participate and provide written informed consent. Exclusion criteria were: (1) comorbid severe psychiatric disorders or cognitive impairment; (2) comorbid other malignant tumors; or (3) critical illness or life expectancy <3 months. Following established guidelines for exploratory factor analysis, a minimum sample size of 5–10 participants per item is recommended (Li et al., 2025c). With 28 items in the BCSAS, the required sample size was calculated as 28 × 10 = 280 participants. To account for an estimated 10% invalid (or incomplete) response rate and to ensure adequate statistical power, we therefore aimed to recruit at least 308 participants. In fact, we successfully recruited 550 participants, thus exceeding our target and providing a robust sample for analysis.

#### 2.3.2 Instruments

The survey consisted of a general information questionnaire and the Chinese version of the BCSAS. The general information questionnaire collected sociodemographic characteristics (e.g., age, education level, occupation, economic status) and clinical information (e.g., time since diagnosis, cancer stage, treatment modalities). The Chinese BCSAS contained 28 items across seven dimensions, rated on a five-point Likert scale (1 = strongly disagree to 5 = strongly agree), with a total score range of 28–140, where higher scores indicate greater stigma.

#### 2.3.3 Data collection

Data were collected using paper-based questionnaires administered face-to-face. Investigators explained the study purpose, confidentiality principles, and instructions before obtaining consent and distributing questionnaires. Investigators remained present during completion to address participants’ questions and ensure data quality. Questionnaires were collected immediately upon completion. Data were entered into Excel using double-entry verification. To ensure quality, all investigators received standardized training. Participants were given clear instructions prior to completion, questionnaires were checked on-site for completeness, and missing responses were promptly addressed. Dual-entry and cross-check procedures minimized data entry errors.

### 2.4 Psychometric evaluation

#### 2.4.1 Item analysis

Item analysis was conducted using high–low group comparison and correlation analysis (Boateng et al., 2018). Based on the total score, the sample was divided into a high-score group and a low-score group using the 27th percentile method, and item-score differences between the two groups were compared (Cappelleri et al., 2014). The 27th percentile was chosen as the cutoff because it is commonly used in psychometrics to maximize discrimination while ensuring sufficient sample size (Kelley, 1939; Rush et al., 2016). Independent-samples *t*-tests were used for normally distributed items, while Mann–Whitney U tests were applied when normality (tested via Kolmogorov–Smirnov) was violated (Chicco et al., 2025). Items with significant t-values or Z-values (*p <* 0.05) in extreme group comparisons were considered to have adequate discriminatory capacity (Cappelleri et al., 2014). Corrected item-total correlations (CITCs) were calculated to assess the relationship between individual items and the overall scale, as well as item-dimension correlations. Items with CITC values ≥0.30 were retained as acceptable, while those with values <0.20 were considered for deletion due to poor discriminatory capacity (Heley et al., 2024; Marlow and Wardle, 2014).

#### 2.4.2 Reliability testing

Reliability was assessed using internal consistency and test–retest reliability. Cronbach’s *α* coefficients were calculated for the overall scale and each dimension, with *α* ≥ 0.70 considered acceptable (Tavakol and Dennick, 2011). For test–retest reliability, 30 patients completed the scale twice with a 2-week interval, and the intraclass correlation coefficient (ICC) was calculated (Cenit-García et al., 2024).

#### 2.4.3 Validity testing

Validity testing included content and construct validity. Content validity was evaluated by the same six experts involved in cultural adaptation (see Section 2.2.2): four nursing psychology specialists and two breast surgery specialists, all meeting the following criteria: (1) mastery of relevant professional knowledge; (2) more than 10 years of work experience (or more than 5 years for those with doctoral degrees); and (3) intermediate or higher professional titles. Experts rated the relevance of each item on a four-point Likert scale (1 = not relevant, 2 = somewhat relevant, 3 = quite relevant, 4 = highly relevant). The item-level content validity index (I-CVI) and scale-level content validity index (S-CVI) were computed, with I-CVI ≥ 0.78 and S-CVI ≥ 0.90 considered acceptable (Imran et al., 2024). Construct validity was examined using CFA to test the original seven-factor, 28-item model. Model fit was evaluated with the following criteria: *χ*^2^/df < 3.0, RMSEA < 0.08, CFI > 0.90, TLI > 0.90, and SRMR < 0.08 (Nguyen et al., 2025). If the initial model did not achieve adequate fit, modification indices (MI > 10) were consulted for theory-consistent adjustments, such as correlating error terms within the same dimension (Whittaker, 2012). Items with factor loadings < 0.50 or cross-loadings > 0.40 were considered for deletion (Lin and Wu, 2014). Convergent validity was assessed using average variance extracted (AVE ≥ 0.50) and composite reliability (CR ≥ 0.70). Discriminant validity was supported if the square root of AVE for each dimension exceeded the inter-construct correlations (Cheung et al., 2024). If convergent or discriminant validity criteria were not met, theoretical considerations and modification indices guided model refinement, including item deletion or merging of similar dimensions, until an empirically and theoretically acceptable final model was achieved.

#### 2.4.4 Network analysis

Exploratory network analysis was incorporated as a complementary method to provide additional insights into item relationships beyond traditional latent variable models (Borsboom, 2017; Ozkok et al., 2019). While CFA assumes local independence (items uncorrelated after controlling for latent factors), network analysis allows examination of direct item-to-item relationships, which may reveal culturally-specific patterns not anticipated in the original model. Network findings were interpreted as descriptive information about stigma phenomenology rather than as evidence requiring structural modifications.

A partial correlation network was constructed using a Gaussian Graphical Model (GGM) with regularization (Epskamp et al., 2018). Centrality indices (Strength, Betweenness, Closeness) identified potentially influential items, and the Louvain algorithm explored natural item clustering patterns (Han et al., 2024). Bootstrap procedures (*n =* 1,000) assessed network stability, with correlation stability coefficients (CS-coefficient) > 0.25 indicating acceptable stability (Feng et al., 2025). This exploratory approach complemented CFA findings to inform clinical understanding, while the seven-factor structure validated through CFA remained the primary psychometric model.

### 2.5 Statistical analysis

All analyses were performed using SPSS 26.0 for descriptive statistics and reliability testing, AMOS 29.0 for confirmatory factor analysis, and R 4.5.0 for network analysis. Continuous variables were described using mean ± standard deviation (M ± SD) or median with interquartile range (IQR), while categorical variables were summarized as frequencies and percentages. All tests were two-tailed, with *p <* 0.05 considered statistically significant. Missing data were handled using pairwise deletion.

## 3 Results

### 3.1 Sample characteristics

A total of 550 questionnaires were distributed, and 500 valid responses were collected, yielding an effective response rate of 90.91%. All participants were female breast cancer patients, with ages ranging from 29 to 85 years (mea*n =* 58.15 ± 10.58). The majority of participants had a primary school education (39.8%), while the remaining sociodemographic and clinical characteristics are summarized in Table 1. The mean total score of the Chinese version of the BCSAS was 85.29 ± 15.56. Subscale scores were as follows: concealability, 22.54 ± 5.84; discrimination, 14.37 ± 3.14; altered self-image/self-concept, 16.03 ± 4.36; family disruption, 9.22 ± 3.17; social attribution, 9.28 ± 2.76; prejudice, 10.27 ± 2.83; and origin, 3.57 ± 1.90. Prior to item analysis, the distribution of all 28 items was examined for normality. As shown in Table 2, most items demonstrated skewness and kurtosis values within the acceptable range of −2 to +2, indicating approximate normality. Only ORIG1 (skewness = 1.917, kurtosis = 3.222) and ORGI2 (skewness = 1.783, kurtosis = 3.085) exhibited mild deviations from normality. For these two items, Mann–Whitney U tests were applied in the high–low group comparisons to ensure robustness of the results.

**Table 1:** General characteristics of the participants (*n =* 500).

Variable	Category	*n*	%
Age	58.15 ± 10.58	–	–
Marital status	Single	62	12.4
	Married	387	77.4
	Widowed	51	10.2
Medical insurance	Self-paid	15	3.0
	Urban resident insurance / New rural cooperative insurance	254	50.8
	Urban employee insurance	231	46.2
Education level	Primary school	199	39.8
	Junior high school / Technical secondary school	135	27.0
	Senior high school / Vocational school	75	15.0
	College and above	91	18.2
Employment status	Full-time	75	15.0
	Unemployed	29	5.8
	Retired	188	37.6
	Others (e.g., housewives, no employment)	208	41.6
Pathological type	Invasive carcinoma, special type	75	15.0
	Invasive carcinoma, no special type	425	85.0
Disease status	Under treatment	164	44.1
	Recurrence	208	55.9
TNM stage	Stage I	26	5.2
	Stage II	192	38.4
	Stage III	110	22.0
	Stage IV	172	34.4
Family history	Yes	54	10.8
	No	446	89.2
Type of surgery	Breast-conserving surgery	10	2.0
	Modified radical mastectomy	412	82.4
	Others (e.g., neoadjuvant chemotherapy)	78	15.6
Total score	85.29 ± 15.56	–	–
Concealability	22.54 ± 5.84	–	–
Discrimination	14.37 ± 3.14	–	–
Altered self-image/self-concept	16.03 ± 4.36	–	–
Family disruption	9.22 ± 3.17	–	–
Social attributions	9.28 ± 2.76	–	–
Prejudices	10.27 ± 2.83	–	–
Origin	3 (3, 3)	–	–

Values are presented as *n* (%) unless otherwise indicated. Age and scale scores are expressed as mean ± standard deviation (M ± SD). Origin is expressed as median (P25, P75). TNM, Tumor–Node–Metastasis.

**Table 2:** Skewness, kurtosis, and normality assessment of the chinese version of the breast cancer stigma scale (BCSAS) items.

Item	Skewness	Kurtosis	Normality judgment
CONC1 (I downplay or minimize my condition in front of some people)	0.009	−1.387	Approx. normal
CONC2 (I regret having told some people that I have breast cancer)	0.261	−1.377	Approx. normal
CONC3 (In certain situations, I feel embarrassed to talk about having breast cancer)	0.396	−1.324	Approx. normal
CONC4 (In some situations I am embarrassed to say that I have breast cancer)	0.225	−1.185	Approx. normal
CONC5 (5. I make an effort to hide or disguise physical changes resulting from breast cancer)	0.381	−1.221	Approx. normal
CONC6 (If I think that I have cancer in my body, I feel disgusted)	0.386	−1.310	Approx. normal
CONC7 (I do not like, or tend to avoid, participating in groups or activities organized by other breast cancer patients)	0.386	−1.146	Approx. normal
DISC1 (I do not like or avoid participating in groups or activities where I have to be with other people with cancer)	−0.935	0.020	Approx. normal
DISC2 (My breast cancer has a negative or limiting effect on me in my work)	−0.495	−1.125	Approx. normal
DISC3 (I feel uncomfortable with the stares, morbidness, or curiosity of some people)	−0.536	−1.026	Approx. normal
DISC4 (Since I was diagnosed with breast cancer, I feel that I have experienced a loss in my social roles)	−0.397	−1.304	Approx. normal
ASISC1 (Since being diagnosed with breast cancer, hair loss or physical sequelae are a significant concern)	0.434	−1.493	Approx. normal
ASISC2 (At times I have found it difficult to say and/or hear the word cancer)	0.397	−1.500	Approx. normal
ASISC3 (I often feel afraid or worried because I feel in danger because of cancer)	0.346	−1.483	Approx. normal
ASISC4 (I feel I am not the same as I was before breast cancer)	0.249	−1.521	Approx. normal
ASISC5 (Having cancer has marked a before and after in my life)	0.236	−1.517	Approx. normal
FADI1 (Having breast cancer harms sexual relations)	0.653	−1.199	Approx. normal
FADI2 (The diagnosis of breast cancer has led to changes in relationships within the extended family)	0.533	−1.301	Approx. normal
FADI3 (The diagnosis of breast cancer has led to a negative impact on the relationship with my partner)	0.550	−1.411	Approx. normal
SOAT1 (Some people who know about my breast cancer make me feel uncomfortable with their attitudes or behaviors)	−0.745	−1.451	Approx. normal
SOAT2 (I feel that some people are uncomfortable with or avoid interacting with me because of my breast cancer, which makes me feel unhappy)	−0.697	−1.521	Approx. normal
SOAT3 (I dislike when others avoid mentioning or talking about the word “cancer.”)	−0.531	−1.725	Approx. normal
SOAT4 (I find it hard to face the fact that I may have difficulty or be unable to be a mother in the future because of cancer)	−0.745	−1.451	Approx. normal
PREJ1 (I dislike when some people treat me differently because of my breast cancer)	0.071	−1.358	Approx. normal
PREJ2 (I worry about how my disease affects the people who care for me)	0.077	−1.081	Approx. normal
PREJ3 (I do not like that some people feel sorry for me)	0.032	−1.561	Approx. normal
ORIG1 (I believe that some of my behaviors or experiences in life may be related to my breast cancer)	1.917	3.222	Deviates from normal
ORGI2 (I believe that having breast cancer was a wake-up call for me and prompted me to change some aspects of my life and self)	1.783	3.085	Deviates from normal

Normality judgment was based on skewness (acceptable range: −2 to +2) and kurtosis (acceptable range: −7 to +7). Items ORIG1 and ORGI2 deviated from normality, while all other items were approximately normal.

### 3.2 Cultural adaptation and pilot testing results

During the translation and cultural adaptation process, modifications were made based on expert feedback, the original author’s suggestions, discussions within the research team, and the findings of the pilot survey. The main revisions were as follows:

Expert feedback: For item SOAT4, our initial literal translation was: *“I find it difficult to face the fact that I may have difficulty or be unable to become a mother.”* Experts suggested modifying it to: *“I find it difficult to face the fact that, due to cancer, I may have difficulty or be unable to breastfeed my child in the future.”* After group discussion, the research team agreed that the expert’s version more comprehensively captured the loss of the maternal role as a social factor. The original author also endorsed this modification, and the expert’s suggestion was therefore adopted.

Author feedback: The original author reviewed the back-translated Chinese version of the BCSAS and provided comments and revisions to ensure conceptual and semantic equivalence across languages. The detailed modifications are presented in Table 3.

**Table 3:** Translation and cultural adaptation of the breast cancer stigma assessment scale (BCSAS): original author feedback and revised Chinese items.

Translation adaptation (initial version)	Evaluation by the original scale author	Revised items (Chinese version)
CONC1: I conceal or avoid talking about my illness to some people.	Avoid talking does not have the same meaning in Spanish as minimize (to minimize is to make it seem that it is less serious or that you are better off than you really are).	I downplay or minimize my condition in front of some people.
CONC3: 1. I do not feel like mentioning anything about my breast cancer in some occasion.	We avoid using borderline expressions (such as not at all, never, always, etc) in order to let the participants express the degree on the likert scale.	In certain situations, I feel embarrassed to talk about having breast cancer.
CONC7: I do not find it enjoyable and will avoid taking part in any communities or group activities held by other breast cancer patients.	“Not finding fun” in Spanish would imply participation, avoidance would imply excluding oneself from that activity out of rejection.	I do not like, or tend to avoid, participating in groups or activities organized by other breast cancer patients.
DISI4: I do not feel as good as normal people since I got breast cancer.	“As valid” in Spanish refers to the possibility of fulfilling social roles; “as good” would also imply moral aspects and the question refers to social roles. “As normal” in Spanish could suggest a prejudice of the researcher about “abnormality” of the patients, and could be stigmatizing in the sense of “deviation/alteration of the norm,” which in Spain would be discriminatory language. If it is not appropriate to the context, avoid using the word “normal” in the wording.	Since I was diagnosed with breast cancer, I feel that I have experienced a loss in my social roles.
ASISC1: Since I got breast cancer, the major problem I have been facing is hair loss or other physical sequelae.	In Spanish, “una preocupación importante” is not the same as “la mayor preocupación.” Some women indicated that the biggest concern was surviving cancer or leaving their children orphaned. Physical appearance was very important, but not necessarily the most important. The degree of importance in Spanish is indicated by the Likert scale rating.	Since being diagnosed with breast cancer, hair loss or physical sequelae are a significant concern.
FADI2: Breast cancer gives rise to poor relationship between parents/children.	In Spain, the family includes, in addition to parents and children, grandparents, siblings, uncles, aunts, uncles, nephews, cousins who may see their roles altered by caring for women. On the other hand, the relationship is altered, but not necessarily for the worse. We consider it appropriate to extend to family relationships and keep “altering” rather than “worsening” if this is also appropriate to their context.	The diagnosis of breast cancer has led to changes in relationships within the extended family.
FADI3: Breast cancer causes a failed conjugal relationship.	In Spain, it is considered that the relationship can be negatively affected, in crisis, and this can be stigmatising for women, without the relationship failing definitively or breaking up. The degree of negative disturbance can be marked by the participant with the Likert scale.	The diagnosis of breast cancer has led to a negative impact on the relationship with my partner.
SOAT1: I have always been beset by the attitude and behaviors of whom know my breast cancer	In the Spanish version we avoid terms such as “siempre” which could be interpreted in a reductionist way. The intensity of discomfort with attitudes and behaviors is defined by the participants with the Likert scale.	Some people who know about my breast cancer make me feel uncomfortable with their attitudes or behaviors.
SOAT2: 2. I have noticed that my breast cancer makes some people feel uncomfortable and evade me.	“I have noticed” does not imply a negative connotation in Spanish, as they can notice it and not find it annoying, and therefore, it is not stigmatising.	I feel that some people are uncomfortable with or avoid interacting with me because of my breast cancer, which makes me feel unhappy.
SOAT3: I do not like people deliberately avoid mentioning or hearing the word “cancer”	“Deliberately” was not used in English because it would imply bad intention on the part of the person omitting the word. Please assess whether it has this meaning in your context.	I dislike when others avoid mentioning or talking about the word “cancer.”
PREJ1: I do not want to be discriminated because of breast cancer	“discriminated” in Spanish is often used for negative treatment, hence “tratar diferente” was used because positive treatment of people without cancer such as condescension, pity or privilege were also stigmatising for the Spanish participants.	I dislike when some people treat me differently because of my breast cancer.
ORIG1: I think it is my lifestyle or life condition that accounts for my breast cancer.	“Lifestyle” is used in Spanish in relation to healthy habits; we used “forma de ser” because the participants had related the causes of the illness to ways of reacting, behaving, being in relation to experiences in their biography, which implies more categories than lifestyle.	I believe that some of my behaviors or experiences in life may be related to my breast cancer.
ORGI2: I think breast cancer is a reminder to me that I need to switch certain aspects of myself and my life.	In Spanish we used the past tense so that the meaning would be the same for women in the active phase of the disease and survivors.	I believe that having breast cancer was a wake-up call for me and prompted me to change some aspects of my life and self.

This table presents examples of the translation–adaptation process for the BCSAS. Column 1 lists the preliminary Chinese translation (initial draft), Column 2 summarizes the original author’s comments on linguistic or cultural nuances, and Column 3 provides the revised Chinese items after integrating expert consensus and author feedback.

### 3.3 Preliminary validation of the scale

#### 3.3.1 Item analysis

A total of 28 items were subjected to item analysis (see Table 4). First, participants were divided into high-score (*n =* 135) and low-score (*n =* 135) groups using the 27% cutoff method based on the total score, and item mean differences between the two groups were examined. Normality tests indicated that most items followed an approximately normal distribution and were therefore analyzed using independent-sample *t*-tests. Items ORIG1 and ORGI2 deviated from normality and were analyzed using the Mann–Whitney U test. Results showed that all items had *t* values ≥ 3.0 (*p <* 0.001), while ORIG1 (U = 7191.50, Z = −3.307, *p* = 0.001) and ORGI2 (U = 6929.50, Z = −3.765, *p <* 0.001) also demonstrated significant group differences. These findings indicate that all items possessed satisfactory discriminative validity.

**Table 4:** Item analysis results of the breast cancer stigma assessment scale (*n =* 270).

Item	High group (*n =* 135) M ± SD	Low group (*n =* 135) M ± SD	*t*	df	*p*	Mean diff.	95% CI of diff.
CONC1	4.25 ± 0.86	2.96 ± 1.06	11.05	268	<0.001	1.296	1.065–1.527
CONC2	4.27 ± 0.77	2.66 ± 0.96	15.31	268	<0.001	1.615	1.407–1.822
CONC3	4.08 ± 0.96	2.46 ± 0.76	15.37	268	<0.001	1.622	1.414–1.830
CONC4	4.08 ± 0.76	2.77 ± 0.86	13.29	268	<0.001	1.311	1.117–1.505
CONC5	4.07 ± 0.87	2.62 ± 0.85	13.87	268	<0.001	1.444	1.239–1.649
CONC6	4.07 ± 0.97	2.57 ± 0.82	13.75	268	<0.001	1.504	1.288–1.719
CONC7	4.13 ± 1.00	2.76 ± 0.78	12.44	268	<0.001	1.363	1.147–1.579
DISC1	4.24 ± 0.79	3.39 ± 0.98	7.86	268	<0.001	0.852	0.639–1.065
DISC2	4.24 ± 0.74	3.03 ± 1.06	10.89	268	<0.001	1.207	0.989–1.426
DISC3	4.24 ± 0.71	3.18 ± 1.02	9.98	268	<0.001	1.067	0.856–1.277
DISC4	4.04 ± 0.60	3.04 ± 1.17	8.84	268	<0.001	1.000	0.777–1.223
ASISC1	3.93 ± 1.29	2.84 ± 1.05	7.60	268	<0.001	1.089	0.807–1.371
ASISC2	3.94 ± 1.27	2.74 ± 1.03	8.54	268	<0.001	1.200	0.923–1.477
ASISC3	4.16 ± 1.17	2.82 ± 1.04	9.93	268	<0.001	1.341	1.075–1.607
ASISC4	4.16 ± 1.09	2.82 ± 1.04	10.37	268	<0.001	1.341	1.086–1.595
ASISC5	4.01 ± 1.22	2.99 ± 1.04	7.42	268	<0.001	1.022	0.751–1.293
FADI1	4.10 ± 1.23	2.61 ± 0.91	11.38	268	<0.001	1.496	1.237–1.755
FADI2	3.99 ± 1.27	2.64 ± 0.89	10.07	268	<0.001	1.341	1.079–1.603
FADI3	3.94 ± 1.27	2.64 ± 1.04	9.16	268	<0.001	1.296	1.018–1.575
SOAT1	2.84 ± 0.55	1.90 ± 1.00	9.51	268	<0.001	0.933	0.740–1.127
SOAT2	2.93 ± 0.38	1.76 ± 0.97	13.02	268	<0.001	1.170	0.993–1.348
SOAT3	2.88 ± 0.47	1.70 ± 0.96	12.90	268	<0.001	1.185	1.004–1.366
SOAT4	2.85 ± 0.53	1.93 ± 1.00	9.44	268	<0.001	0.919	0.727–1.110
PREJ1	4.21 ± 1.04	3.11 ± 1.06	8.58	268	<0.001	1.096	0.845–1.348
PREJ2	4.20 ± 0.97	3.14 ± 0.90	9.32	268	<0.001	1.059	0.835–1.283
PREJ3	4.27 ± 1.06	3.08 ± 1.09	9.05	268	<0.001	1.185	0.927–1.443
ORIG1	–	–	Mann–Whitney U	7191.50	0.001	–	–
ORGI2	–	–	Mann–Whitney U	6929.50	<0.001	–	–

Values are presented as mean ± standard deviation (M ± SD). High group = upper 27% of the total score (*n =* 135); Low group = lower 27% (*n =* 135). Independent-samples *t*-test was applied for normally distributed items; Mann–Whitney U test was applied for items ORIG1 and ORGI2 due to non-normal distribution. All items demonstrated significant discrimination (*p <* 0.01).

Second, corrected item–total correlations (CITC) were examined (see Table 5). Most items demonstrated CITC values above the 0.30 threshold, with items such as FADI1 (harms sexual relations) (*r* = 0.576) and PREJ2 (worry about disease’s impact on caregivers) (*r* = 0.543) showing relatively strong correlations with the total score. Although ORIG1 (belief about behavioral/experiential origins) (*r* = 0.222) and ORGI2 (cancer as wake-up call for life changes) (*r* = 0.308) displayed weaker correlations, both were still within the acceptable range. Additionally, Cronbach’s *α* values did not increase substantially when any single item was deleted, indicating that item retention was reasonable. In summary, results from both the extreme group comparisons and the item–total correlation analyses demonstrated that all 28 items had good discriminative power and internal consistency. Therefore, all items were retained for subsequent reliability, validity, and network analyses.

**Table 5:** Item–total statistics of the breast cancer stigma assessment scale (*n =* 500).

Item	CITC	Cronbach’s *α* if item deleted
CONC1	0.395	0.888
CONC2	0.517	0.885
CONC3	0.547	0.885
CONC4	0.491	0.886
CONC5	0.517	0.885
CONC6	0.503	0.886
CONC7	0.532	0.885
DISC1	0.329	0.889
DISC2	0.442	0.887
DISC3	0.401	0.888
DISC4	0.309	0.890
ASISC1	0.460	0.887
ASISC2	0.474	0.886
ASISC3	0.498	0.886
ASISC4	0.530	0.885
ASISC5	0.402	0.888
FADI1	0.576	0.884
FADI2	0.515	0.885
FADI3	0.481	0.886
SOAT1	0.358	0.889
SOAT2	0.418	0.888
SOAT3	0.444	0.887
SOAT4	0.348	0.889
PREJ1	0.475	0.886
PREJ2	0.543	0.885
PREJ3	0.446	0.887
ORIG1	0.222	0.892
ORGI2	0.308	0.890

CITC, Corrected item–total correlation. Items with CITC < 0.30 (ORIG1, ORGI2) may indicate weak correlation with the overall construct. “Cronbach’s *α* if item deleted” shows the reliability coefficient when the item is removed. Overall scale Cronbach’s *α* = 0.890.

#### 3.3.2 Reliability analysis

The overall Cronbach’s *α* coefficient of the Chinese version of the BCSAS was 0.890. Internal consistency across dimensions was also satisfactory, with *α* values of 0.870 for Concealability, 0.765 for Discrimination, 0.763 for Altered Self-Image/Self-Concept, 0.833 for Family Disruption, 0.705 for Social Attribution, 0.799 for Prejudice, and 0.810 for Origin, all exceeding the recommended threshold of 0.70.

#### 3.3.3 Validity analysis

##### 3.3.3.1 Content validity

Based on expert ratings, the S-CVI was 0.98, and the I-CVI ranged from 0.88 to 1.00, both of which exceeded the acceptable thresholds (S-CVI ≥ 0.90; I-CVI ≥ 0.78).

##### 3.3.3.2 Confirmatory factor analysis

**Figure 1:**
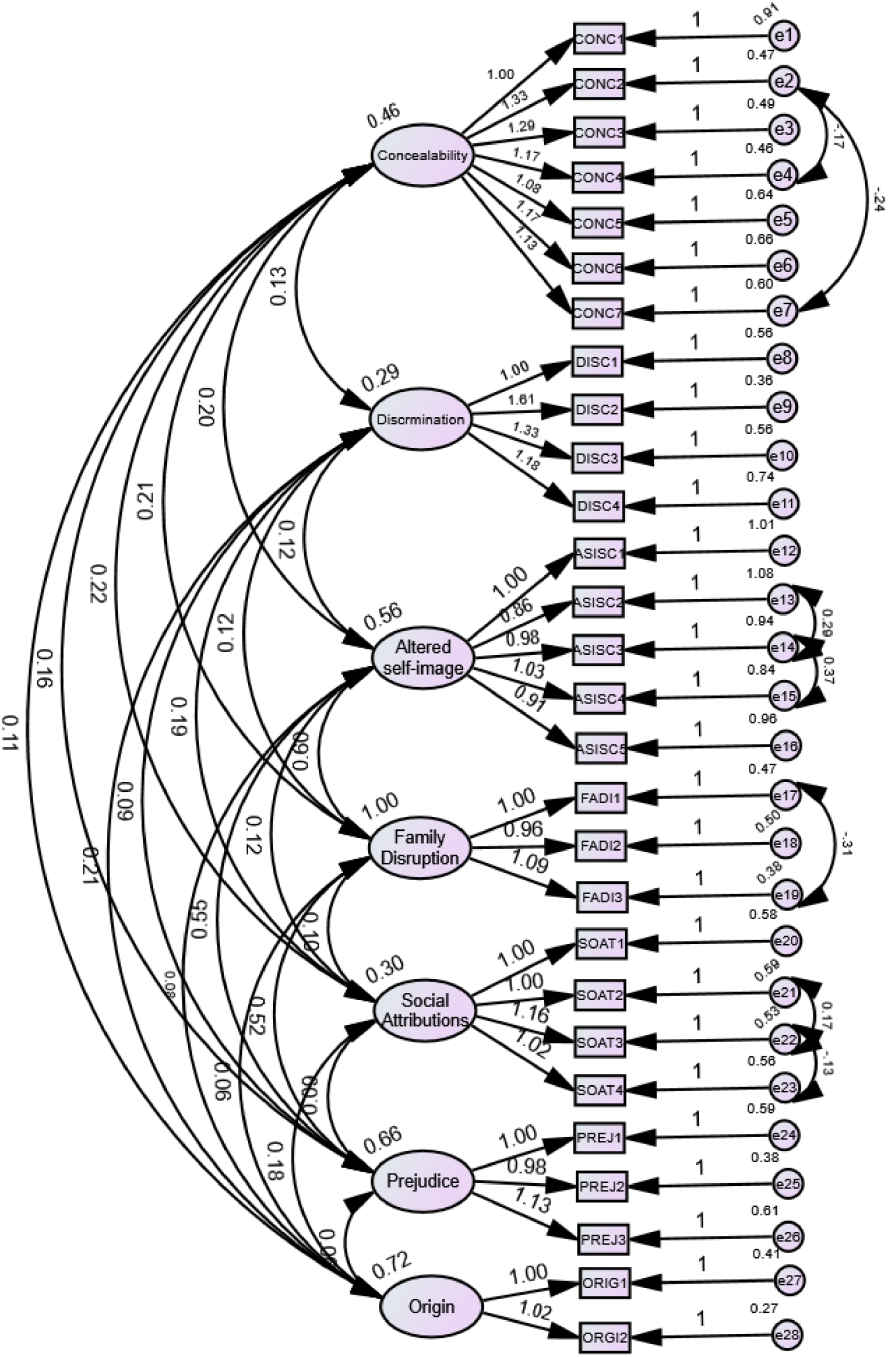
Confirmatory factor analysis (CFA) model of the seven-factor Chinese version of the Breast Cancer Stigma Assessment Scale (C-BCSAS) after modification. The figure presents the structural equation model of the seven-factor, 28-item structure after theory-consistent modifications based on modification indices. Ovals represent the seven latent factors: (1) Concealability, (2) Discrimination, (3) Altered Self-Image/Self-Concept, (4) Family Disruption, (5) Social Attribution, (6) Prejudice, and (7) Origin. Rectangles represent observed items (28 total). Single-headed arrows from factors to items show standardized factor loadings (all *λ* > 0.50, all *p <* 0.001). Small circles represent measurement errors (e1–e28). Curved double-headed arrows between error terms (e2-e7, e2-e4, e13-e14, e17-e19, e21-e22, e22-e23) represent correlated errors added based on modification indices (MI > 10) to account for shared method variance or similar item wording. Model fit: *χ*2/df = 4.446, CFI = 0.829, TLI = 0.800, GFI = 0.848, AGFI = 0.809, RMSEA = 0.083 (90% CI = 0.079–0.088). Despite marginal fit, this structure was retained to preserve theoretical integrity and enable cross-cultural comparison.

CFA was conducted to test the original seven-factor structure of the BCSAS in the Chinese sample. The initial model fit indices indicated suboptimal fit: *χ*^2^(329) = 1688.896, *p <* 0.001; *χ*^2^/df = 5.133; RMSEA = 0.091 (90% CI = 0.087–0.095, PCLOSE < 0.001); CFI = 0.791; TLI = 0.760; GFI = 0.817; AGFI = 0.774. All indices fell short of commonly recommended criteria (*χ*^2^/df < 3, RMSEA < 0.08, CFI/TLI > 0.90, GFI > 0.90). After incorporating correlated error terms suggested by modification indices (e2↔e7, e2↔e4, e13↔e14, e17↔e19, e21↔e22, e22↔e23), the model fit improved: *χ*^2^/df = 4.446, RMSEA = 0.083 (90% CI = 0.079–0.088, PCLOSE < 0.001), CFI = 0.829, TLI = 0.800, GFI = 0.848, AGFI = 0.809, but remained below the recommended thresholds. While these indices indicated suboptimal fit, they provided context for exploring item relationships through complementary network analysis (Figure 1).

Despite marginal global fit indices, all standardized factor loadings were greater than 0.50, ranging from 0.540 to 0.846, which met the commonly accepted criteria for convergent validity. Specifically, items such as FADI1 (0.836), PREJ2 (0.808), ORIG1 (0.806), and ORGI2 (0.846) exhibited the highest loadings within their respective factors, indicating strong representativeness. In contrast, SOAT1 (uncomfortable attitudes from others) (0.540), DISC1 (avoids cancer-related activities) (0.584), and CONC1 (downplays condition) (0.597) showed relatively lower, but still acceptable, factor loadings. All loadings were statistically significant (*p <* 0.001) (see Table 6). Reliability analysis demonstrated satisfactory internal consistency (Cronbach’s *α* = 0.890; dimension-specific *α* = 0.712–0.876) and test–retest reliability (ICC = 0.825), supporting the retention of the seven-factor, 28-item structure for the Chinese version of the BCSAS.

**Table 6:** Standardized factor loadings of the BCSAS (*n =* 500).

**Dimension**	**Item**	**Standardized loading**	**R**^**2**^ **(SMC)**
Concealability (F1)	CONC1	0.597	0.356
	CONC2	0.757	0.573
	CONC3	0.788	0.620
	CONC4	0.715	0.512
	CONC5	0.697	0.486
	CONC6	0.714	0.510
	CONC7	0.694	0.481
Discrimination (F2)	DISC1	0.584	0.341
	DISC2	0.822	0.676
	DISC3	0.692	0.479
	DISC4	0.596	0.356
Altered self-image/self-concept (F3)	ASISC1	0.573	0.328
	ASISC2	0.548	0.301
	ASISC3	0.676	0.457
	ASISC4	0.682	0.465
	ASISC5	0.576	0.331
Family Disruption (F4)	FADI1	0.836	0.698
	FADI2	0.730	0.532
	FADI3	0.637	0.406
Social Attributions (F5)	SOAT1	0.540	0.292
	SOAT2	0.677	0.458
	SOAT3	0.767	0.588
	SOAT4	0.593	0.352
Prejudice (F6)	PREJ1	0.718	0.515
	PREJ2	0.808	0.652
	PREJ3	0.749	0.561
Origin (F7)	ORIG1	0.806	0.649
	ORGI2	0.846	0.715

All factor loadings are standardized and significant at *p <* 0.001. R^2^ = Squared Multiple Correlation.

### 3.4 Network analysis results

**Figure 2:**
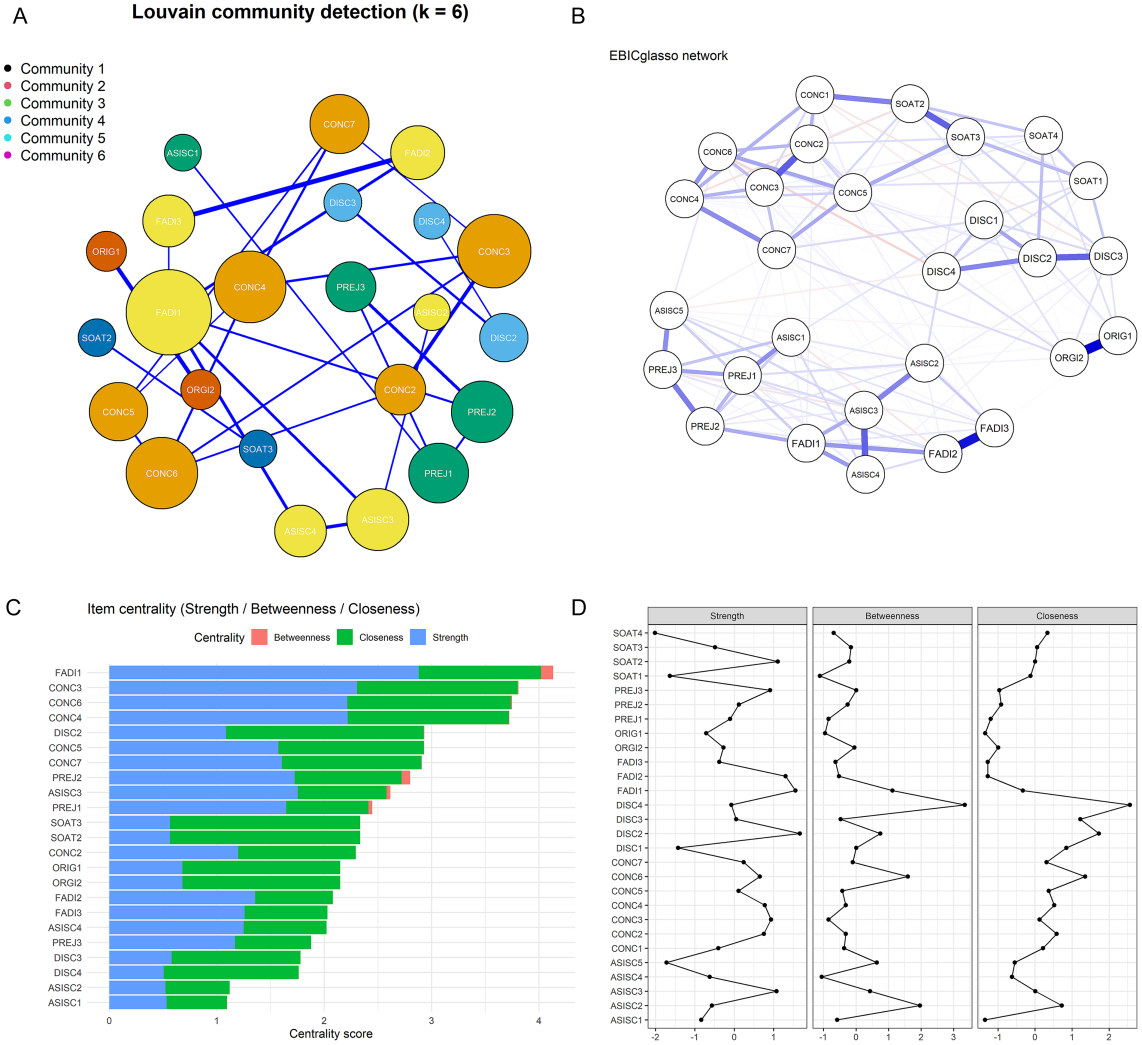
Network analysis of the Chinese version of the Breast Cancer Stigma Scale (BCSAS). **(A)** Network structure with Louvain community detection. Nodes represent items, edges represent partial correlations estimated via EBICglasso, and colors indicate community membership. **(B)** Nonparametric bootstrap results for edge weights (95% confidence intervals), indicating the stability of pairwise associations between items. **(C)** Centrality indices (strength, betweenness, closeness) of individual items, highlighting their relative importance in the network. **(D)** Bootstrapped centrality stability plot from the bootnet package, showing the robustness of strength, betweenness, and closeness indices under case-dropping bootstrap.

To complement the CFA findings and provide additional insights into stigma phenomenology, exploratory network analysis was conducted. Three centrality indices—Strength, Betweenness, and Closeness—were calculated to evaluate the network characteristics of the 28 items (see Table 7). Results indicated that FADI1 (“Having breast cancer harms sexual relations”) was the most prominent item, with the highest Strength centrality (2.88), suggesting it had the strongest connections with other stigma experiences and served as the core node of the network (Figure 2C). Items CONC3 (“I feel embarrassed to talk about having breast cancer”), CONC4 (“I am embarrassed to say that I have breast cancer”), and CONC6 (“If I think that I have cancer in my body, I feel disgusted”) also demonstrated relatively high Strength values (2.22–2.31), highlighting the central role of concealment and disclosure-related concerns in the stigma experience of Chinese breast cancer patients.

**Table 7:** Network centrality and community detection of the Chinese version of the breast cancer stigma scale (*n =* 500).

Item	Strength	Betweenness	Closeness	Community
FADI1	2.881901	0.112554	1.136166	4
CONC3	2.305998	0.007937	1.494968	1
CONC4	2.219274	0.002886	1.500613	1
CONC6	2.215644	0.007937	1.524008	1
ASISC3	1.753727	0.034632	0.826842	4
PREJ2	1.72599	0.077922	0.995845	3
PREJ1	1.647134	0.034632	0.765272	3
CONC7	1.606665	0.001443	1.299975	1
CONC5	1.572949	0.001443	1.355964	1
FADI2	1.357589	0	0.722751	4
FADI3	1.259972	0	0.77111	4
ASISC4	1.25153	0	0.770572	4
CONC2	1.19898	0	1.096016	1
PREJ3	1.170705	0	0.707662	3
DISC2	1.086456	0.004329	1.840849	2
ORIG1	0.68123	0	1.467932	6
ORGI2	0.68123	0	1.467932	6
DISC3	0.579556	0	1.200472	2
SOAT2	0.565082	0	1.769654	5
SOAT3	0.565082	0	1.769654	5
ASISC1	0.533453	0	0.561512	3
ASISC2	0.523561	0	0.597083	4
DISC4	0.506899	0	1.255213	2

Strength reflects the sum of absolute connections of each node; betweenness indicates the extent to which a node bridges different parts of the network; closeness represents the proximity of a node to all other nodes in the network; community was identified using Louvain community detection (numbers correspond to modularity-based clusters).

Regarding Betweenness centrality, FADI1 (sexual relations impact) (0.113), PREJ2 (“I worry about how my disease affects the people who care for me”) (0.078), and ASISC3 (“I feel afraid or worried because I feel in danger because of cancer”) (0.035) scored the highest, indicating their potential bridging roles across different stigma dimensions and their function in facilitating interdimensional connections. In terms of Closeness centrality, FADI1 (sexual relations impact) again ranked the highest (1.136), underscoring its overall importance within the stigma network. Community detection using the Louvain algorithm (Figure 2A) identified six major clusters (Community 1–6): Community 1 included Concealability items (CONC2–CONC7: regret disclosure, embarrassment, hiding physical changes, body disgust, avoiding patient groups), reflecting strong internal consistency within this dimension; Community 2 included Discrimination items (DISC2–DISC4: work limitations, uncomfortable stares, social role loss); Community 3 comprised Prejudice items (PREJ1–PREJ3: differential treatment, worry about caregivers, dislike pity) along with ASISC1 (hair loss concerns); Community 4 clustered Altered Self-Image/Self-Concept items (ASISC2–ASISC4: difficulty with cancer word, fear and danger, changed self, life turning point) together with Family Disruption items (FADI1–FADI3: sexual relations, extended family changes, partner relationship), suggesting a close empirical association between body image changes and family-related concerns in the Chinese sample, though this co-occurrence does not necessarily indicate these domains are conceptually identical; Community 5 consisted of Social Attribution items (SOAT2: others avoiding me; SOAT3: avoiding cancer word); and Community 6 contained Origin items (ORIG1: behavioral causation; ORGI2: wake-up call).

Furthermore, nonparametric bootstrapping confirmed the robustness of edge weights and centrality indices (Figures 2B,D). Confidence intervals of edge weights demonstrated a stable network structure (Figure 2B), while stability analyses of node centrality revealed that the correlation stability coefficient (CS-coefficient) for Strength exceeded the acceptable threshold (>0.25), indicating reliable identification of core nodes (Figure 2D). Taken together, the network analysis revealed complex patterns of inter-item interactions and identified several key items—particularly sexual relations impact (FADI1) and concealment-related items (CONC3, CONC4, CONC6) as central to the stigma network among Chinese breast cancer patients. These findings provide important implications for understanding the psychological mechanisms underlying stigma and identifying potential intervention priorities, while complementing the theory-driven seven-factor structure established through CFA (Figure 1).

## 4 Discussion

### 4.1 Psychometric properties of the Chinese version of the breast cancer stigma assessment scale

The Chinese version of the BCSAS demonstrated satisfactory reliability among Chinese female breast cancer patients and survivors, though structural validity showed room for improvement. This study represents the first effort to introduce and validate the Chinese version of the BCSAS in a sample of breast cancer patients from western Liaoning, China. Following the Brislin translation model, a standardized forward–backward translation procedure was strictly adhered to, ensuring linguistic and semantic equivalence of the scale. The original seven-factor, 28-item structure was retained to preserve theoretical coherence and enable cross-cultural comparison. Confirmatory factor analysis revealed fit indices of *χ*^2^/df = 4.446, CFI = 0.829, TLI = 0.800, and RMSEA = 0.083. While these indices fall slightly below commonly cited thresholds, it is important to contextualize model fit in cross-cultural validation studies. In large samples (*n* > 500), even minor residual covariances can produce statistically significant *χ*^2^ values and lower incremental fit indices, without necessarily indicating fundamental theoretical or structural problems (Chen, 2007; Marsh et al., 2004). Moreover, cross-cultural factors such as response style tendencies, linguistic nuances in translated items, and heterogeneity in clinical and sociodemographic variables can contribute to reduced fit indices while the underlying construct structure remains valid (Davidov et al., 2014).

Critically, all standardized factor loadings substantially exceeded 0.50 (range: 0.540–0.846) and were statistically significant (*p <* 0.001), supporting convergent validity at the item level. Reliability analysis confirmed strong internal consistency (overall *α* = 0.890; dimension-specific *α* = 0.712–0.876) and good temporal stability (ICC = 0.825). Content validity was excellent (S-CVI = 0.98), with expert consensus that all 28 items were culturally relevant and appropriate. Collectively, these psychometric indicators demonstrate that the C-BCSAS is a reliable and valid instrument for assessing breast cancer stigma in Chinese contexts. The observed fit indices likely reflect genuine cultural variation in how stigma dimensions co-occur and correlate—for example, stronger associations between self-image and family concerns in collectivistic cultures—rather than construct invalidity. Following best practices in cross-cultural measurement, we prioritized conceptual and content equivalence alongside acceptable (rather than perfect) statistical fit (Beaton et al., 2000; van de Vijver and Leung, 2021).

### 4.2 Network analysis as exploratory complement: insights without structural modification

Network analysis was incorporated as a complementary exploratory method to examine inter-item relationships from an alternative perspective, without serving as a basis for revising the scale structure. This methodological decision aligns with the principle that exploratory network findings should inform understanding rather than override established theoretical frameworks (Frost, 2020).

The network analysis revealed several important phenomenological insights into the organization of stigma experiences in Chinese breast cancer patients. Family disruption FADI1 emerged as the most central node (Strength = 2.88), suggesting it is the most interconnected stigma experience among Chinese patients. This finding aligns with cultural expectations in collectivistic societies where family-related concerns are paramount (Cui et al., 2021; Jin et al., 2021). Alongside family concerns, items related to concealment and self-image (CONC3, CONC4, CONC6) also showed high centrality, highlighting the importance of disclosure management and identity concerns in the stigma experience. Most notably, community detection identified empirical clustering that partially diverged from the theoretical structure, with self-image and family disruption items grouping together in the network.

Despite this empirical clustering, we did not interpret it as evidence that these dimensions should be merged. According to Goffman’s foundational stigma theory and Fujisawa’s cancer-specific framework, altered self-concept represents the individual and identity-based essence of stigma (intrapersonal process), while family disruption reflects relational and contextual dimensions of stigma (interpersonal process). These are conceptually distinct mechanisms that may co-occur empirically—particularly in collectivistic cultures—but operate through different psychological pathways (Link and Phelan, 2001).

The strong network connectivity between self-image and family items indicates that these experiences are phenomenologically intertwined in Chinese patients’ lived experience, a pattern supported by multiple empirical studies. Cui and Wang (2024) showed that stigma influenced depressive symptoms through social constraints (i.e., the suppression of emotional expression due to fear of family or societal judgment), while Xu et al. (2021) revealed through qualitative research how breast cancer survivors experienced stigma in relation to family role adjustments, parent–child interactions, and emotional communication. These studies demonstrate that in Chinese collectivistic culture, stigma experiences are deeply embedded in family relationships—but this does not mean that self-perception and family dynamics are the same construct.

The distinction is critical: While Chinese women may experience disruptions in self-image and family relationships as closely linked (high empirical correlation), the underlying mechanisms differ fundamentally. Altered self-concept involves internalized shame, changed body image, and threatened identity (individual psychological processes), whereas family disruption involves changes in communication patterns, role performance, and relational harmony (interpersonal social processes). Merging these domains would conflate different levels of analysis and obscure which aspect of stigma is most amenable to specific interventions—for example, cognitive restructuring for self-concept issues versus family psychoeducation for relational disruption.

Given these theoretical considerations, we retained the distinction between these dimensions despite their strong empirical association in the Chinese sample. Future research should examine whether this pattern replicates in independent Chinese samples and whether the strength of association between self-image and family domains differs systematically across cultures (e.g., comparing collectivistic versus individualistic societies using multi-group CFA). Future research should also incorporate variables such as anticipated family evaluation, filial obligation, and face concerns to further validate these pathways, and examine how such mechanisms vary across age groups, levels of family support, and broader social contexts.

### 4.3 Cross-cultural adaptation tensions: balancing statistical fit and theoretical integrity

A central challenge in this study was navigating the tension between achieving optimal statistical fit and preserving the theoretical and cross-cultural integrity of the BCSAS. Cross-cultural validation research inherently involves balancing two objectives: (1) cultural appropriateness (emic validity)—ensuring the instrument resonates with local cultural meanings; and (2) cross-cultural comparability (etic validity)—maintaining structural equivalence to enable valid international comparisons (van de Vijver and Leung, 2021). The observed fit indices (*χ*^2^/df = 4.446, CFI = 0.829, TLI = 0.800, RMSEA = 0.083) can be interpreted as either model misspecification requiring structural revision, or as genuine cultural variation in stigma phenomenology that should be described rather than “corrected.” We adopted the latter interpretation for several reasons.

Modifying the factor structure to optimize fit would enhance local statistical performance but undermine cross-cultural comparability—a primary rationale for adapting an existing instrument rather than developing a new one. The BCSAS was selected because its theoretical framework (Fujisawa and Hagiwara, 2015; Goffman, 1963) and dimensional structure have been validated internationally. Substantial structural modifications would preclude meta-analyses and cross-national research essential for understanding global patterns of cancer stigma. Moreover, the theoretical framework underlying the BCSAS remains conceptually valid in Chinese contexts. The distinction between intrapersonal stigma (self-concept) and interpersonal stigma (social/family disruption) continues to be relevant even if these dimensions co-occur more strongly in collectivistic than individualistic cultures. Wu et al. (2023) identified five core attributes of breast cancer stigma in Chinese patients that align closely with the BCSAS dimensions, supporting the relevance of the original structure. The question is not whether these constructs exist in Chinese culture, but whether their interrelationships differ across cultures—a question best addressed through measurement invariance testing rather than structural revision in a single sample (Wu et al., 2023).

The relationship between the C-BCSAS and Bu et al.'s (2022) Chinese-developed Breast Cancer Stigma Scale (BCSS) merits clarification (Bu et al., 2022). Both demonstrated satisfactory reliability (C-BCSAS *α* = 0.890; BCSS *α* = 0.86). However, the instruments differ substantially in theoretical scope. The BCSS is a brief indigenous instrument (15 items, four dimensions) focusing primarily on impaired self-image, social isolation, discrimination, and internalized stigma. Notably, approximately 40% of BCSS items focus specifically on postoperative and appearance-related experiences, which may limit applicability to women at different disease stages. Moreover, the BCSS does not explicitly assess family disruption, origin attributions, or social attribution—dimensions identified as theoretically important in contemporary psychosocial oncology research.

In contrast, the C-BCSAS provides a theoretically comprehensive framework grounded in Goffman's (1963) stigma theory and Fujisawa and Hagiwara's (2015) cancer-specific model. Its seven-dimension, 28-item structure encompasses both intrapersonal dimensions (altered self-concept, concealment) and interpersonal dimensions (family disruption, discrimination, prejudice, social attribution), as well as cognitive-attributional processes (origin beliefs). Importantly, the C-BCSAS includes a dedicated Family Disruption dimension that explicitly addresses the family-centered concerns repeatedly identified as central to Chinese patients’ stigma experiences (Cui et al., 2021; Xu et al., 2021; Yeung et al., 2019). This comprehensive coverage makes the C-BCSAS suitable for both culturally grounded research in China and international comparative research, while maintaining theoretical integrity necessary to advance global understanding of cancer stigma mechanisms.

### 4.4 Limitations

Despite rigorous translation procedures and comprehensive psychometric evaluation, several limitations must be acknowledged. First, the sample was drawn exclusively from western Liaoning Province, which may restrict the generalizability of the findings to other regions or cultural contexts within China, such as southern provinces or areas with more distinct cultural variations. Second, the cross-sectional design limited our ability to capture the dynamic changes of stigma across different treatment stages; longitudinal studies are warranted to explore the temporal evolution of stigma experiences in breast cancer patients. Third, while the scale demonstrated excellent reliability (*α* = 0.890, ICC = 0.825) and content validity (S-CVI = 0.98), CFA fit indices fell slightly below commonly cited thresholds (*χ*^2^/df = 4.446, CFI = 0.829, TLI = 0.800, RMSEA = 0.083). As discussed in Section 4.1, these indices should be contextualized within cross-cultural validation research, where factors such as large sample sizes, response style tendencies, and cultural variation in construct organization can affect fit without indicating fundamental structural problems. All standardized factor loadings substantially exceeded 0.50 (range: 0.540–0.846, *p <* 0.001), supporting item-level validity. Following best practices in cross-cultural measurement, we prioritized conceptual and content equivalence alongside acceptable (rather than perfect) statistical fit. Future research should: (1) conduct measurement invariance testing across Chinese and international samples to formally assess cross-cultural equivalence; (2) examine whether fit varies across subgroups defined by age, disease stage, or treatment modality; and (3) explore whether the strength of associations between self-image and family domains differs systematically across collectivistic versus individualistic cultures. Fourth, the decision to retain the original seven-factor structure prioritizes theoretical integrity and cross-cultural comparability over local fit optimization. As discussed in Section 4.3, this approach enables rigorous international comparisons and meta-analyses while preserving the BCSAS’s established theoretical framework. Researchers seeking brief, culturally optimized assessment may prefer indigenous measures, while those requiring comprehensive, cross-culturally comparable measurement will find the C-BCSAS more suitable. Finally, as this study relied on self-reported data, potential social desirability bias cannot be ruled out; future research should integrate behavioral observations or clinician assessments to complement self-report measures.

## 5 Conclusion

This study successfully developed a Chinese version of the Breast Cancer Stigma Assessment Scale (C-BCSAS) through rigorous cross-cultural adaptation procedures. The C-BCSAS demonstrates satisfactory reliability (Cronbach’s *α* = 0.890; ICC = 0.825) and excellent content validity (S-CVI = 0.98), supporting its use as a valid instrument for assessing stigma experiences among Chinese women with breast cancer and survivors. By retaining the original seven-factor, 28-item structure, the C-BCSAS preserves theoretical fidelity to Fujisawa and Hagiwara’s established cancer stigma framework, enabling meaningful cross-cultural comparisons while remaining culturally appropriate for Chinese contexts. This culturally adapted yet structurally equivalent instrument provides a valuable tool for both clinical assessment of stigma-related psychological distress and international collaborative research on cancer stigma, contributing to the advancement of culturally sensitive psycho-oncology care and global cancer stigma research.

## Data Availability

The raw data supporting the conclusions of this article will be made available by the authors, without undue reservation.

## Glossary

BCSAS: Breast Cancer Stigma Assessment Scale
CNN: China’s National Cancer Center
CFA: Confirmatory Factor Analysis
RMSEA: Root Mean Square Error of Approximation
GFI: Goodness of Fit Index
TLI: Tucker-Lewis Index
CR: Composite Reliability
AVE: Average Variance Extracted
S-CVI: Scale-level Content Validity Index
I-CVI: Item-level Content Validity Index
TNM: Tumor, Node, Metastasis
HRS: Health-Related Stigma
PTSD: Post-Traumatic Stress Disorder
SPSS: Statistical Package for the Social Sciences
CONC: Concealability
ASISC: Altered Self-Image/Self-Concept
FADI: Family Disruption
SOAT: Social Attributions
PREJ: Prejudices
DISC: Discrimination
ORIG: Origin
